# Prevalence, Outcome, and Prevention of Congenital Cytomegalovirus Infection in Neonates Born to Women With Preconception Immunity (CHILd Study)

**DOI:** 10.1093/cid/ciac482

**Published:** 2022-06-19

**Authors:** Daniele Lilleri, Beatrice Tassis, Lorenza Pugni, Andrea Ronchi, Carlo Pietrasanta, Arsenio Spinillo, Alessia Arossa, Cristian Achille, Patrizia Vergani, Sara Ornaghi, Silvia Riboni, Paolo Cavoretto, Massimo Candiani, Gerarda Gaeta, Federico Prefumo, Nicola Fratelli, Anna Fichera, Michele Vignali, Allegra Barbasetti Di Prun, Elisa Fabbri, Irene Cetin, Anna Locatelli, Sara Consonni, Simona Rutolo, Elena Miotto, Valeria Savasi, Maria Di Giminiani, Antonella Cromi, Sandro Binda, Loretta Fiorina, Milena Furione, Gabriela Cassinelli, Catherine Klersy, for the CHILd Study Group, Stefania Piccini, Valentina Marrazzi, Giulia Muscettola, Paola Zelini, Piera d’Angelo, Marica De Cicco, Daniela Cirasola, Federica Zavaglio, Lea Testa, Claudia Ballerini, Rebecca Stachetti, Marta Ruggiero Fondazione, Federica De Liso, Annalisa Cavallero, Isadora Vaglio Tessitore, Maria Luisa Ventura, Mirko Pozzoni, Camilla Merlo, Giulia Rivetti, Vania Spinoni, Gaia Belloni, Camilla Querzola, Marta Pessina, Elisa Ligato, Alice Zavatta, Marta Balconi, Serena Mussi, Patrizia Biraghi, Selene Cammarata, Fabio Ghezzi, Massimo Agosti, Laura Pellegrinelli, Cristina Galli, Valeria Primache

**Affiliations:** Microbiologia e Virologia, Fondazione IRCCS Policlinico San Matteo, Pavia, Italy; Fondazione IRCCS Ca’ Granda Ospedale Maggiore Policlinico, Milan, Italy; Fondazione IRCCS Ca’ Granda Ospedale Maggiore Policlinico, Milan, Italy; Fondazione IRCCS Ca’ Granda Ospedale Maggiore Policlinico, Milan, Italy; Fondazione IRCCS Ca’ Granda Ospedale Maggiore Policlinico, Milan, Italy; Department of Clinical Sciences and Community Health, University of Milan, Milan, Italy; Ostetricia e Ginecologia, Fondazione IRCCS Policlinico San Matteo, Pavia, Italy; Ostetricia e Ginecologia, Fondazione IRCCS Policlinico San Matteo, Pavia, Italy; Neonatologia e Terapia intensiva neonatale, Fondazione IRCCS Policlinico San Matteo, Pavia, Italy; Fondazione Monza Brianza per il Bambino e la sua Mamma Onlus c/o Ospedale San Gerardo, Università Milano-Bicocca Scuola di Medicina e Chirurgia, Monza, Italy; Fondazione Monza Brianza per il Bambino e la sua Mamma Onlus c/o Ospedale San Gerardo, Università Milano-Bicocca Scuola di Medicina e Chirurgia, Monza, Italy; Fondazione Monza Brianza per il Bambino e la sua Mamma Onlus c/o Ospedale San Gerardo, Università Milano-Bicocca Scuola di Medicina e Chirurgia, Monza, Italy; Gynecology and Obstetrics Department, IRCCS San Raffaele Hospital and University, Milan, Italy; Gynecology and Obstetrics Department, IRCCS San Raffaele Hospital and University, Milan, Italy; Gynecology and Obstetrics Department, IRCCS San Raffaele Hospital and University, Milan, Italy; ASST Spedali Civili di Brescia and University of Brescia, Brescia, Italy; ASST Spedali Civili di Brescia and University of Brescia, Brescia, Italy; ASST Spedali Civili di Brescia and University of Brescia, Brescia, Italy; ASST Fatebenefratelli-Sacco, Ospedale Macedonio Melloni, Milan, Italy; Dipartimento di Scienze Biomediche per la Salute, Università degli Studi di Milano, Milan, Italy; ASST Fatebenefratelli-Sacco, Ospedale Macedonio Melloni, Milan, Italy; Dipartimento di Scienze Biomediche per la Salute, Università degli Studi di Milano, Milan, Italy; Dipartimento di Ostetricia e Ginecologia, Ospedale dei Bambini Vittore Buzzi, Università di Milano, Milan, Italy; Dipartimento di Ostetricia e Ginecologia, Ospedale dei Bambini Vittore Buzzi, Università di Milano, Milan, Italy; ASST Brianza (Ospedali di Carate e Vimercate), Vimercate, Italy; ASST Brianza (Ospedali di Carate e Vimercate), Vimercate, Italy; ASST Monza, Ospedale di Desio, Desio, Italy; ASST Monza, Ospedale di Desio, Desio, Italy; Unit of Obstetrics and Gynecology, ASST Fatebenefratelli-Sacco, Milan, Italy; Department of Biological and Clinical Sciences, University of Milan, Milan, Italy; Unit of Obstetrics and Gynecology, ASST Fatebenefratelli-Sacco, Milan, Italy; Department of Biological and Clinical Sciences, University of Milan, Milan, Italy; Ospedale Del Ponte, Università dell’Insubria, Varese, Italy; Dipartimento di Scienze Biomediche per la Salute, Università degli Studi di Milano, Milan, Italy; Microbiologia e Virologia, Fondazione IRCCS Policlinico San Matteo, Pavia, Italy; Microbiologia e Virologia, Fondazione IRCCS Policlinico San Matteo, Pavia, Italy; Microbiologia e Virologia, Fondazione IRCCS Policlinico San Matteo, Pavia, Italy; Epidemiologia clinica e Biostatistica, Fondazione IRCCS Policlinico San Matteo, Pavia, Italy

**Keywords:** human cytomegalovirus, congenital infection, nonprimary infection, preconception immunity

## Abstract

**Background:**

Human cytomegalovirus (HCMV) is the leading infectious cause of congenital disabilities. We designed a prospective study to investigate the rate, outcome, and risk factors of congenital CMV (cCMV) infection in neonates born to immune women, and the potential need and effectiveness of hygiene recommendations in this population.

**Methods:**

The study was composed of 2 sequential parts: an epidemiology (part 1) and a prevention (part 2) study. Performance of part 2 depended upon a cCMV rate >0.4%. Women enrolled in part 1 did not receive hygiene recommendations. Newborns were screened by HCMV DNA testing in saliva and cCMV was confirmed by urine testing.

**Results:**

Saliva swabs were positive for HCMV DNA in 45/9661 newborns and cCMV was confirmed in 18 cases. The rate of cCMV was .19% (95% confidence interval [CI]: .11–.29%), and 3 out of 18 infants with cCMV had symptoms of CMV at birth. Age, nationality, occupation, and contact with children were similar between mothers of infected and noninfected newborns. Twin pregnancy (odds ratio [OR]: 7.2; 95% CI: 1.7–32.2; *P* = .037) and maternal medical conditions (OR: 3.9; 95% CI: 1.5–10.1; *P* = .003) appeared associated with cCMV. Given the rate of cCMV was lower than expected, the prevention part of the study was cancelled.

**Conclusions:**

Newborns from women with preconception immunity have a low rate of cCMV, which appears to be mostly due to reactivation of the latent virus. Therefore, serological screening in childbearing age would be pivotal to identify HCMV-seropositive women, whose newborns have a low risk of cCMV.

**Clinical trials registration:**

www.clinicaltrials.gov (NCT03973359).

## Teaser

Congenital HCMV infection occurs in 0.19% of immune mothers, among which it appears mostly due to reactivation of the latent virus. Serological screening in childbearing age is pivotal to identify HCMV-seropositive women, who have a low risk of congenital CMV.

In developed countries, human cytomegalovirus (HCMV) is the leading cause of congenital infections, which may result in neurocognitive and psychomotor delay, hearing loss, speech and language disabilities, behavioral disorders, and visual impairment [1]. Approximately 0.6% of newborns are HCMV-congenitally infected [2] and, among these, 20–25% are symptomatic at birth or will develop long-term sequelae [3, 4], with a substantial public health impact.

Preconception immunity does not provide complete protection against non-primary maternal infection (ie, reactivation of the latent virus or reinfection with a new strain) and vertical transmission. A positive correlation between maternal seroprevalence and rate of congenital HCMV infection (cCMV) was observed, ranging from 0.3% in populations with 30% seroprevalence to 2% in populations with 98% seroprevalence [2, 5]. Whether cCMV after maternal nonprimary infection is the consequence of a reactivation or a reinfection remains undefined, notwithstanding that previous studies have argued that maternal superinfection could play a significant role [6, 7].

A meta-analysis reported similar percentages of symptomatic infants following either primary or nonprimary infections [8]. Finally, it has been estimated that nonprimary maternal infections account for the majority of HCMV-related hearing deficits [9]. Presently, although actively sought, an HCMV vaccine is not available [10].

The most important route of acquisition of primary HCMV infection is through contact with young children, as they actively shed the virus in saliva and urine. A recent controlled study showed a significant reduction in the seroconversion rate from 7.6% to 1.2% in seronegative women caring for toddlers who received HCMV counseling compared with women who did not receive any information [11].

A study conducted in a highly immune population (Brazil) reported that a significantly higher number of transmitter compared with nontransmitter mothers cared for toddlers [7]. Therefore, HCMV-shedding toddlers may represent a risk of reinfection for seropositive pregnant women as well.

We designed the present study (NCT03973359) to investigate the rate and outcome of cCMV in neonates born to immune women in northern Italy, as well as the potential need and effectiveness of hygiene recommendations in this population.

## METHODS

### Study Design

The Congenital Human cytomegalovirus Infection in Lombardy (CHILd) Study was a prospective study composed of 2 sequential parts. Part 1 (epidemiology study) was aimed at investigating the incidence and outcome of cCMV in neonates born to women with preconception immunity. Part 2 (prevention study) was designed to investigate the effectiveness of hygiene measures for the prevention of cCMV in this population. We planned to include 10 000 women in part 1 and 13 523 women in part 2, with newborns examined for cCMV. Sample size would have been either confirmed or recalculated based on an interim analysis planned after the examination of 5000 newborns. The study was approved by the Ethics Committee of Fondazione IRCCS Policlinico San Matteo (Comitato Etico Pavia, P-20170011101) and participants signed a written informed consent.

For part 1, women with HCMV serology compatible with a remote infection were enrolled either at the beginning of pregnancy (≤13 weeks’ gestation) or at delivery, provided that a medical record of HCMV serology at 13 weeks of gestation or less or before pregnancy was available (although not recommended, serological screening for HCMV infection is usually performed). In addition, no HCMV-related hygiene recommendation is given to HCMV-seropositive women in the participating centers as part of normal antenatal care. Blood samples were collected from women enrolled at 13 weeks of gestation or less for retrospective additional testing in case of cCMV. Saliva swabs from newborns were collected by the hospital staff within 72 hours of life and shipped to the central laboratory for HCMV DNA testing. In case of a positive result, the samples were retested: if positivity was confirmed, newborn urine was requested and tested for HCMV DNA (within 3 weeks after birth). Only when HCMV DNA was detected in urine was the newborn was diagnosed with cCMV. In case urine samples were not obtained, congenital infection was confirmed by HCMV DNA detection on the dry blood spots collected at birth for screening of metabolic and genetic disorders. Blood, urine, dry saliva, and vaginal swabs of the mothers of infected newborns were collected after delivery. Part 2 was designed as a continuation of part 1, with the addition of providing hygiene information at enrollment (which had to be performed only at ≤13 weeks’ gestation).

Infants with documented cCMV were clinically assessed at diagnosis and at 3, 6, and 12 months of age. Maternal and newborn data were collected in an electronic case report form (REDCap platform). Details on the study population, sample size and interim analysis, inclusion and exclusion criteria, and laboratory testing are reported in the Supplementary Material (and Supplementary Figure 1). The same protocol was adopted in each center.

### Objective and Measures

The primary objective for part 1 was the prevalence and clinical outcomes of cCMV in neonates born to women with preconception immunity. The primary objective for part 2 was the efficacy of hygiene counseling in reducing cCMV in the same population (as compared with part 1). Secondary objectives for part 1 were to identify possible serological parameters associated with nonprimary infection and potential risk factors for cCMV.

### Statistical Analysis

Continuous data are described with the median and interquartile range (IQR) and compared with the Mann-Whitney *U* test. Categorical data are reported as counts and percentages and compared with the chi-square test. The rate of cCMV is expressed as a percentage and exact binomial 95% confidence interval (CI). The odds ratio (OR) of potential risk factors for congenital infection and its 95% CI were computed using univariable logistic regression. Analyses were performed using GraphPad Prism software (version 8; GraphPad Software, Inc).

## RESULTS

### Primary Endpoint: Number and Outcome of Infected Newborns

**Figure 1.**
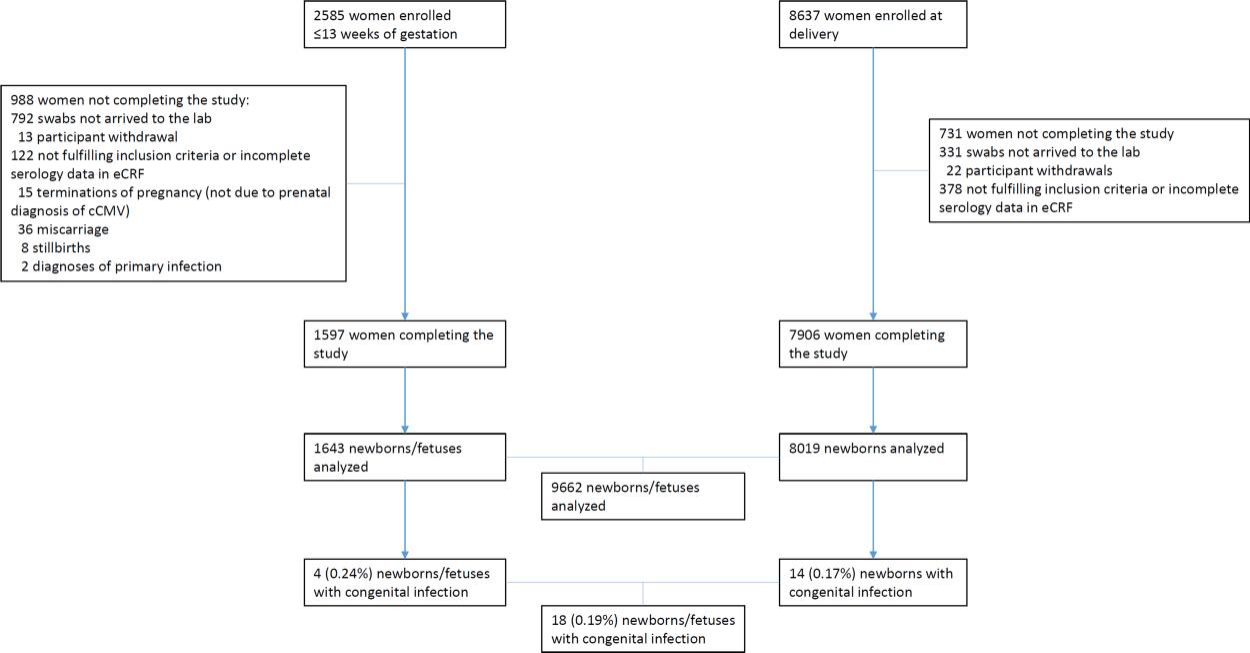
Study population and rates of cCMV infection. Abbreviations: cCMV, congenital cytomegalovirus; eCRF, electronic Case Report Form.

Between September 2017 and October 2020, 11 222 pregnant women were enrolled (Figure 1): 8637 at delivery and 2585 at 13 weeks of gestation or less. Of them, 9503 women completed the study (7906 enrolled at delivery and 1597 enrolled at ≤13 weeks’ gestation). At the end of the study, 9661 newborns and 1 fetus terminated in utero for severe disease from 9503 pregnancies were examined: 18 congenitally infected newborns/fetuses were observed, with a rate of .19% (95% CI: .11–.29%). Congenital infection rate was similar among women enrolled at 13 weeks of gestation or less or at delivery (Figure 1).

Among the 9661 newborns examined, 45 were positive for HCMV DNA in saliva collected within 72 hours of birth, and congenital infection was confirmed in 17 newborns by repeat testing of that saliva sample and by detection of virus in newborn urine collected 3 weeks after birth for 15 newborns or in dry blood spots for 2 newborns whose urine was either not collected due to the parents’ refusal or it was collected 32 days after birth.

In 11 newborns with a positive saliva screen and a positive result on repeat testing of that saliva specimen, congenital infection was not confirmed because HCMV DNA was not detected in a urine sample. In 17 newborns with a positive saliva screen with less than 250 copies/mL of HCMV DNA, retesting of that sample was negative; urine was not tested and congenital infection was not confirmed (see Table 1 for the infected newborns and Supplementary Table 1 for results of the 45 newborns with positive saliva screening).

**Table 1.** Characteristics of the 18 Infected Newborns

Subject	HCMV DNA, Copies/mL		Symptoms at Birth	Treatment	Sequelae at 1 Year
	Saliva Swab	Urine			
MG 371-403 Twin2	72 425	36 460	No	No	No
MG 371-426	86 571	50 351	No	No	No
MG 371-1965	3 241 918	60 199 569	No	No	No
SA 365-63^a^	2 147 137	NA	No	No	No
SA 365-81^a^	1359	3 324 726	No	No	No
PV 363-921	149 950	1 496 529	No	No	No
PV 363-999	69 333	7 346 775	No	No	No
PV 363-1104^b^	NA	NA	ToP	NA	NA
DE 367-145 Twin1	1491	97 562	No	No	No
DE 367-145 Twin2	512	55 690	No	No	No
DE 367-227	1324	107 738	No	No	No
SR 373-516	55 634	41 026 281	Jaundice, left ear positive aABR, low platelet and neutrophil count, abnormal cerebral US and MRI, severe early onset fetal growth restriction	GCV/VGCV	Reduced somatic growth and neurodevelopmental delay
VI 369-79	406 408	484 721	No	No	No
VI 369-143	5215	111 285 558	Petechieae, low platelet count, epatosplenomegaly, elevated AST	GCV/VGCV	Mild cognitive delay
VI 369-156	4749	4161	No	No	No
BU 364-284	48 941	32 802 624	No	No	No
SC 372-470	35 566	1423	No	No	No
MB 368-1103	121 212	3 827 428	No	No	No

Abbreviations: aABR, automated Auditory Brainstem Response; AST, aspartate transaminase; GCV/VGCV, ganciclovir/valganciclovir; HCMV, human cytomegalovirus; MRI, magnetic resonance imaging; NA, not available; ToP, termination of pregnancy; US, ultrasound.

Congenital infection confirmed retrospectively on Guthrie card (urine of SA 365-81 was collected 32 days after birth).

Congenital infection diagnosed on amniotic fluid after US abnormalities and subsequent ToP. Further details of the severely symptomatic case number SR-373-516 can be found in a recently published report [12].

**Figure 2.**
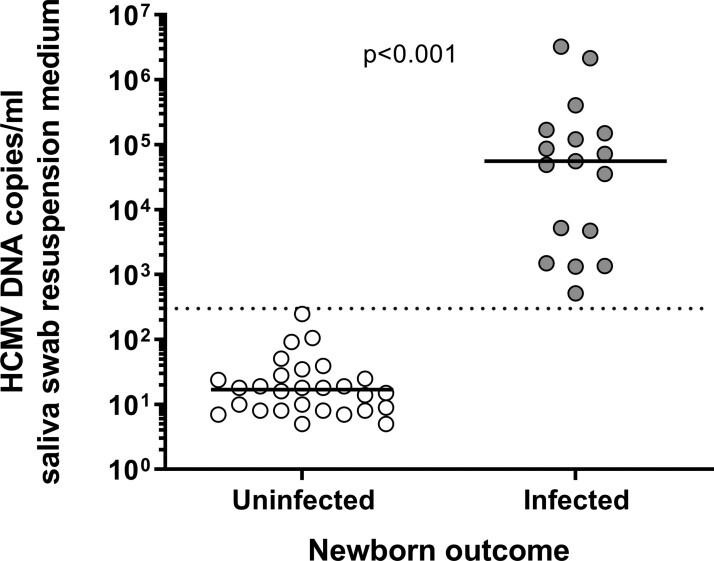
HCMV DNA levels in saliva swabs of infected or uninfected (positive in saliva but negative in urine) newborns with HCMV DNA positive saliva swab at birth. Mann-Whitney *U* test was used for statistical analysis. Abbreviation: HCMV, human cytomegalovirus.

Levels of HCMV DNA in saliva swabs of the 17 confirmed cases were above 500 copies/mL in resuspension medium (median level: 5.6 × 10^4^; range: 0.5 × 10^3^–3.2 × 10^6^ copies/mL), whereas levels of HCMV DNA in saliva swabs of the 28 nonconfirmed cases were below 250 copies/mL (median level: 1.7 × 10^1^; range: 0.5 × 10^1^–2.5 × 10^2^ copies/mL) (Figure 2).

One additional case of cCMV was diagnosed by HCMV DNA detection in the amniotic fluid (Table 1): prenatal diagnosis was performed at 21 weeks of gestation because of abnormal findings at ultrasound examination (periventricular hyperechogenicity, corpus callosum hypoplasia). Cordocentesis showed 3 of 4 altered parameters (among viral load, platelet count, HCMV-specific immunoglobulin [Ig] M [IgM], and B2 microglobulin), suggesting symptomatic fetal infection. Fetal magnetic resonance imaging confirmed pathological cerebral findings. The pregnancy was terminated and histological examination showed the following: severe brain damage in the form of multiple necro-inflammatory foci predominantly affecting the cortex, white matter, and germinal matrix, often accompanied by calcifications. Data on the infected newborns are shown in Table 1. Two infected newborns were twins (monochorionic diamniotic gestation), and another infected newborn had a noninfected twin brother (dichorionic diamniotic gestation). Symptomatic congenital infection was observed in 3 of 18 (17%) newborns/fetuses, whereas the other 15 asymptomatic newborns did not show sequelae at 1 year of age.

### Characteristics of the Mothers of Congenitally Infected Children

Data from the 17 mothers with cCMV newborns/fetus are shown in Table 2. No women had HCMV-specific IgM at the beginning of pregnancy. Moreover, a possible periconceptional infection could be definitively be ruled out in 13 of 17 mothers due to previous serology reports showing positive HCMV-IgG and negative HCMV-IgM before the index pregnancy, or high HCMV-IgG avidity on the serum collected at enrollment.

**Table 2.** Characteristics of the Mothers of Congenitally Infected Children

Subject	Concomitant Medical Conditions	Serostatus Before^a^ or at the Beginning of Pregnancy				HCMV DNA, Copies/mL				
		Days After LMP	IgG	IgM	Avidity Index	Days After Delivery	Saliva Swab	Vaginal Swab	Urine	Blood
MG 371-403 (twin pregnancy)	No	−81	Pos	Neg	NA	22	0	24 179	474	0
MG 371-426	Syphilis in treatment	94	Pos	Neg	NA	16	0	243	14	0
MG 371-1965	No	83	Pos	Neg	High	16	435	45 342	192	72
SA 365-63	Thyroid dysfunction	72	Pos	Neg	NA	97	20	0	ND	0
SA 365-81^b^	No	46	Pos	Neg	NA	37	75	9	2670	10
PV 363-921	No	86	Pos	Neg	High	3	0	0	0	0
PV 363-999	Seroconversion for *Toxoplasma gondii*	85	Pos	Neg	High	13	0	2308	180	0
PV 363-1104^c^ (ToP)	No	56	Pos	Neg	High	21 weeks	0	0	845	62
DE 367-145^d^ (twin pregnancy)	No	67	Pos	Neg	NA	18	21	57	1309	0
DE 367-227	Gestational diabetes, sideropenic anemia	−536	Pos	Neg	NA	6	0	0	0	8
SR 373-516	Pre-eclampsia, systemic lupus erythematosus in treatment	71	Pos	Neg	High	2	19	132	0	0
VI 369-79	Gestational diabetes, thyroid dysfunction	53	Pos	Neg	NA	26	296	197	33	0
VI 369-143	Thyroid dysfunction	61	Pos	Neg	High	7	0	18	0	NA
VI 369-156	Mitral valve prolapse	77	Pos	Neg	NA	13	0	48	52	0
BU 364-284^e^	Gestational diabetes, thyroid dysfunction	71	Pos	Neg	High	49	0	84	1289	0
SC 372-470^f^	No	76	Pos	Neg	NA	19	109	3348	0	55
MB 368-1103	No	62	Pos	Neg	High	14	1433	11	7	0

Abbreviations: HCMV, human cytomegalovirus; Ig, immunoglobulin; LMP, last menstrual period; NA, not available; ND, not done; Neg, negative; Pos, positive; ToP, termination of pregnancy.

A minus sign indicates days before LMP.

IgG Pos/IgM Neg in the previous pregnancy (3 years before).

IgG Pos/IgM Neg (IgG 3-fold the cutoff level) in the previous pregnancy (3 years before).

IgG Pos/IgM Neg in the previous pregnancy (3 years before).

Primary infection during the previous pregnancy (2 years before).

IgG Pos/IgM Neg 3 months before pregnancy.

For 9 women (53%), 1 or more concurrent medical conditions during pregnancy were reported (Table 2). After delivery, HCMV DNA was detected in bodily fluids or blood in all but 2 women. Four women enrolled at the beginning of pregnancy had whole-blood samples stored and HCMV DNA testing gave negative results.

In order to attempt to make a distinction between HCMV reinfection or reactivation, genotype-specific IgG response to glycoprotein (g)B and gH was analyzed in 7 of the 17 transmitting women for whom serum samples were available both at the beginning of pregnancy and at delivery (Supplementary Table 2). Genotype-specific IgG to gB was detected in 3 women at the beginning of pregnancy (all anti-gB2/3). The same IgG antibody specificity was detected at delivery. Among the 4 women with no anti-gB genotype–specific IgG antibody at the beginning of pregnancy, 1 woman (VI 369-143) showed the appearance of anti-gB2/3 IgG at delivery. Six of the 7 women had detectable anti-gH genotype–specific IgG antibody at the beginning of pregnancy, and the same genotype specificity was detected at delivery. VI 369-143 showed IgG specific for both gH1 and gH2 genotypes in the 2 time points analyzed. Thus, a potential reinfection with a new HCMV strain was detected in 1 of 7 women tested.

### Risk Factors for Vertical Transmission

The median age of transmitting mothers was 33 years (IQR: 30–38 years), similar to the median age of nontransmitting mothers: 33 years (IQR: 30–37 years). Occupation and nationality (Italian or foreigner) were not significantly different between the 2 groups (data not shown), or was the presence of living children and contact with young children younger than 36 months of age (for family or professional reasons) (Table 3). On the other hand, twin pregnancy was more common in transmitting mothers (OR: 7.2; 95% CI: 1.7–32.2; *P* = .037). Most interestingly, the presence of concomitant medical conditions was more frequent in transmitting than in nontransmitting mothers (OR: 3.9; 95% CI: 1.5–10.1; *P* = .003). Among the specific concomitant pathologies observed, diabetes (OR: 4.1; 95% CI: 1.9–14.5) and thyroid dysfunction (OR: 2.9; 95% CI: .9–8.8) were more commonly associated with cCMV.

**Table 3.** Factors Associated With Congenital Cytomegalovirus Infection

Variables	No. of Women	No. (%) of Women Transmitting HCMV	*P*	Odds Ratio (95% CI)
Presence of living children				
Present	4483	5 (0.11)	.153	.5 (.2–1.3)
Absent	4999	12 (0.24)		
Contact with children <36 months				
Present	3131	6 (0.19)	.802	1.1 (.4–3.0)
Absent	6360	11 (0.17)		
Twin pregnancy				
Present	171	2 (1.17)	.037	7.2 (1.7–32.2)
Absent	9281	15 (0.16)		
Concomitant medical conditions				
Present	2136	9 (0.42)	.003	3.9 (1.5–10.1)
Absent	7346	8 (0.11)		
Diabetes				
Present	469	3 (0.64)	.016	4.1 (1.9–14.5)
Absent	9030	14 (0.16)		
Thyroid dysfunction				
Present	919	4 (0.44)	.053	2.9 (.9–8.8)
Absent	8579	13 (0.15)		
Hypertension				
Present	210	1 (0.48)	.308	2.7 (.4–20.8)
Absent	9286	16 (0.17)		
Autoimmune diseases				
Present	181	1 (0.55)	.230	3.2 (.4–24.5)
Absent	9314	16 (0.17)		
Other				
Present	734	4 (0.54)	.015	3.7 (1.2–11.3)
Absent	8761	13 (0.13)		

Abbreviations: CI, confidence interval; HCMV, human cytomegalovirus.

### Withdrawal of the Prevention Part

The planned interim analysis was conducted after the examination of 5260 newborns. HCMV DNA was detected in the saliva of 31 newborns and congenital infection was confirmed in 12 newborns (.23%; 95% CI: .12–.39%) from 11 mothers (1 twin pregnancy). The 0.4% expected rate of cCMV in the uninformed population was beyond the upper limit of the 95% CI of the observed rate of cCMV. No particular characteristics of transmitting women were identified, since age, nationality, occupation, and close contact with young children were similar between mothers of infected and noninfected newborns. Therefore, data from the interim analysis indicated that the study did not need to proceed to part 2 (prevention part). The final analysis of part 1 (epidemiology) confirmed the interim analysis results and justified the decision to withdraw the prevention part.

## DISCUSSION

Results of the CHILd study show that the prevalence of cCMV among newborns/fetuses from mothers with preconception immunity is .19% (95% CI: .11–.29%) in northern Italy. Maternal age and close contact with young children are not associated with cCMV in this population, whereas the presence of maternal medical conditions during pregnancy is associated.

The prevalence of cCMV in immune mothers of the CHILd study was lower than that reported in immune mothers from the highly seroprevalent (98%) population of Brazil, where the rate of cCMV was 0.5% [13]. On the other hand, our results are close to what was reported in Finland [14], where an overall 0.2% prevalence of cCMV was observed (although the rate of cCMV in immune mothers was not determined) in a population with a seroprevalence rate (70%) similar to that in Italy [15, 16]. In France, an overall 0.37% prevalence of cCMV was reported, with a 0.2% prevalence of cCMV in a subgroup of mothers with known preconception immunity [17] (see Supplementary Table 3 comparing the cCMV frequency in studies assessing maternal seropositivity at the beginning of pregnancy).

We diagnosed cCMV by saliva screening and subsequent confirmation by urine (or dry blood spot) testing. Our analysis found that 28 of 45 (62%) newborns with HCMV DNA–positive saliva were not congenitally infected, confirming that saliva testing alone has a poor positive-predictive value [14, 17, 18]. False-positive saliva had significantly lower viral load, and a cutoff value greater than 2.5 × 10^2^ HCMV DNA copies/mL identified infected newborns, similarly to what was reported by Eventov-Friedman et al [18]. HCMV shedding in genital secretions or breast milk is a potential cause of peripartum or postpartum saliva contamination in noninfected newborns. Therefore, saliva testing alone may overestimate the actual prevalence of cCMV. Although the validation of a viral load cutoff may improve the reliability of saliva testing, currently it should be considered as a preliminary screening to be subsequently confirmed on urine sample.

Among potential maternal risk factors, we did not find a younger age in mothers of cCMV newborns, as instead observed in Brazil [5] and France [17]. Most important, we did not find an association between caring for young children and cCMV. This result contrasts with what was observed in immune mothers in Brazil [7] but is in line with what was reported in France, where contact with young children was associated with cCMV in seronegative but not seropositive mothers [17]. Since young children are the major source of HCMV exposure in seronegative women, this finding suggests that exposure to HCMV and reinfection was unlikely to be the major cause of cCMV in immune mothers of our cohort. Conversely, reactivation of the latent virus may have been the more common cause of cCMV in both the CHILd and the French studies. We previously reported the results of a nested study conducted in a subgroup of pregnant women enrolled [19], showing that contact with children was not associated with nonprimary infection during pregnancy.

Among laboratory testing, HCMV-specific IgM antibodies were not detected at the beginning of pregnancy in the transmitting women. Additionally, HCMV DNA was not detected in the blood of the 4 cases tested.

The detection of new strain-specific serological responses has already been adopted in the attempt to discriminate between HCMV reinfection and reactivation as the cause of cCMV [6, 7]. In our study, only 1 in 7 women examined had serological evidence of reinfection. However, genotype-specific antibody testing may lack sensitivity in detecting reinfections.

The contrasting results observed between European countries and Brazil [7, 13] may be due to the different socioeconomic conditions and HCMV seroprevalence of the 2 populations. Higher exposure may occur in countries with high seroprevalence and, thus, favor the occurrence of reinfections, which instead appear infrequent in Europe.

Instead, we found an association of cCMV with twin pregnancy, as also reported elsewhere [5], and with the presence of comorbidities. The latter finding also suggests that reactivation may be at the basis of cCMV in seropositive women. Conditions such as diabetes [20], which may alter immunological responses [21], immunosuppressive treatments [12], or concurrent infections may have favored HCMV reactivation.

The strength of the CHILd study is the analysis of cCMV frequency in a large cohort of pregnant women with known seropositivity at the beginning of pregnancy. Limitations reside in the low prevalence of cCMV, which made it impossible to identify definite risk factors and to investigate the potential effectiveness of hygiene recommendations in preventing cCMV in seropositive women, and in the lack of direct comparison with the rate of cCMV in pregnant women without preconception immunity. In addition, the relative contribution of reinfection or reactivation was investigated in a small sample, and not finding evidence of infection with a new gB or gH serotype does not completely rule out reinfection.

The major outcome is that the risk for cCMV in immune mothers is quite low (<2 cases in 1000 pregnancies) in Italy and, most likely, in European countries, where the seroprevalence is approximately 70%. This risk appears at least 10 times lower than that observed in seronegative mothers in contact with young children (8 cCMV cases out of 315 seronegative mothers; ie, 2.5%) in a previous study [11]. A 4-fold higher risk for seronegative women to deliver a cCMV newborn was also reported in France [17]. Close contact with young children, the main risk factor for primary infection, is not associated with cCMV in seropositive mothers in high-income countries. This epidemiological evidence suggests that most congenital infections may be due to reactivation of the latent HCMV rather than reinfection with a new strain. Although the study was initially designed to verify the effectiveness of hygienic measures to prevent cCMV in immune mothers, the prevention part of the study was withdrawn due to the actual low prevalence of cCMV, and no clear risk factor was identified to select women who may take advantage of behavioral interventions. However, the lack of association between contact with children and cCMV argues against the potential effectiveness of such measures.

In conclusion, these results support the protective role of maternal preconception immunity in preventing cCMV, thus endorsing the potential effectiveness of vaccination strategies whenever an HCMV vaccine should become available. Finally, preconception screening in childbearing age would be pivotal to identify HCMV-seropositive women, who have a low risk of cCMV. Conversely, the identification, counseling, and prospective monitoring of seronegative pregnant women [22, 23] are even more crucial now that secondary prevention of cCMV through timely intervention with antivirals after primary infection appears to be effective [24], and administration of hyperimmunoglobulin provided promising results [25].

## Supplementary Data

Supplementary materials are available at *Clinical Infectious Diseases* online. Consisting of data provided by the authors to benefit the reader, the posted materials are not copyedited and are the sole responsibility of the authors, so questions or comments should be addressed to the corresponding author.

## Supplementary Material

ciac482_Supplementary_Data

## Notes

***Acknowledgments.*** The authors thank the women participating in this study and the personnel of participating centers. A contract research organization (Consorzio Italiano per la Ricerca in Medicina, Italy) was responsible for the regulatory aspects of the study and verification of source documents.

***Data sharing.*** Access to the eCRF of enrolled women will be granted upon request to the corresponding author.

***Financial support.*** This work was supported by Fondazione Regionale per la Ricerca Biomedica (grant number FRRB 2015-0043).

***Collaborators of the CHILd Study Group.*** Stefania Piccini (Fondazione IRCCS Policlinico San Matteo, Pavia, Italy); Valentina Marrazzi (Fondazione IRCCS Policlinico San Matteo, Pavia, Italy); Giulia Muscettola (Fondazione IRCCS Policlinico San Matteo, Pavia, Italy); Paola Zelini (Fondazione IRCCS Policlinico San Matteo, Pavia, Italy); Piera d’Angelo (Fondazione IRCCS Policlinico San Matteo, Pavia, Italy); Marica De Cicco (Fondazione IRCCS Policlinico San Matteo, Pavia, Italy); Daniela Cirasola (Fondazione IRCCS Policlinico San Matteo, Pavia, Italy); Federica Zavaglio (Fondazione IRCCS Policlinico San Matteo, Pavia, Italy); Lea Testa (Fondazione IRCCS Ca’ Granda Ospedale Maggiore Policlinico, Milano, Italy); Claudia Ballerini (Fondazione IRCCS Ca’ Granda Ospedale Maggiore Policlinico, Milano, Italy); Rebecca Stachetti (Fondazione IRCCS Ca’ Granda Ospedale Maggiore Policlinico, Milano, Italy); Marta Ruggiero Fondazione (IRCCS Ca’ Granda Ospedale Maggiore Policlinico, Milano, Italy); Federica De Liso (Fondazione IRCCS Ca’ Granda Ospedale Maggiore Policlinico, Milano, Italy); Annalisa Cavallero (Università Milano-Bicocca Scuola di Medicina e Chirurgia, Fondazione Monza Brianza per il Bambino e la sua Mamma Onlus c/o Ospedale San Gerardo, Monza, Italy); Isadora Vaglio Tessitore (Università Milano-Bicocca Scuola di Medicina e Chirurgia, Fondazione Monza Brianza per il Bambino e la sua Mamma Onlus c/o Ospedale San Gerardo, Monza, Italy); Maria Luisa Ventura (Università Milano-Bicocca Scuola di Medicina e Chirurgia, Fondazione Monza Brianza per il Bambino e la sua Mamma Onlus c/o Ospedale San Gerardo, Monza, Italy); Mirko Pozzoni (Gynecology and Obstetrics Department, IRCCS San Raffaele Hospital and University, Milan, Italy); Camilla Merlo (ASST Spedali Civili di Brescia and University of Brescia, Italy); Giulia Rivetti (ASST Spedali Civili di Brescia and University of Brescia, Italy); Vania Spinoni (ASST Spedali Civili di Brescia and University of Brescia, Italy); Gaia Belloni (ASST Fatebenefratelli-Sacco, Ospedale Macedonio Melloni, Milano, Italy); Camilla Querzola (Ospedale dei Bambini Vittore Buzzi, Università di Milano, Milano, Italy); Marta Pessina (Ospedale dei Bambini Vittore Buzzi, Università di Milano, Milano, Italy); Elisa Ligato (Ospedale dei Bambini Vittore Buzzi, Università di Milano, Milano, Italy); Alice Zavatta (Ospedale dei Bambini Vittore Buzzi, Università di Milano, Milano, Italy); Marta Balconi (ASST Brianza (Ospedali di Carate e Vimercate), Italy); Serena Mussi (ASST Brianza (Ospedali di Carate e Vimercate), Italy); Patrizia Biraghi (ASST Brianza (Ospedali di Carate e Vimercate), Italy); Selene Cammarata (Unit of Obstetrics and Gynecology, ASST Fatebenefratelli-Sacco, and Department of Biological and Clinical Sciences, University of Milan, Milan, Italy); Sonia Paolucci (Università dell’Insubria, Ospedale Del Ponte, Varese, Italy); Fabio Ghezzi (Università dell’Insubria, Ospedale Del Ponte, Varese, Italy); Massimo Agosti (Università dell’Insubria, Ospedale Del Ponte, Varese, Italy); Laura Pellegrinelli (Dipartimento di Scienze Biomediche per la Salute, Università degli Studi di Milano, Italy); Cristina Galli (Dipartimento di Scienze Biomediche per la Salute, Università degli Studi di Milano, Italy); Valeria Primache (Dipartimento di Scienze Biomediche per la Salute, Università degli Studi di Milano, Italy).
