# Supplementary material for: Prevalence, Outcome, and Prevention of Congenital Cytomegalovirus Infection in Neonates Born to Women With Preconception Immunity (CHILd Study)
Source: Clin Infect Dis. 2022 Jun 19;76(3):513–20. doi: 10.1093/cid/ciac482 (PMC9907511; doi:10.1093/cid/ciac482)
Supplement: ciac482_Supplementary_Data [file ciac482_supplementary_data.pdf]

Supplementary material

Collaborators of the CHILd Study group

Study design

Study population, sample size and interim analysis

Collaborators of the CHILd Study group

Investigator		Affiliation
Stefania	Piccini	Fondazione IRCCS Policlinico San Matteo, Pavia, Italy
Valentina	Marrazzi	Fondazione IRCCS Policlinico San Matteo, Pavia, Italy
Giulia	Muscettola	Fondazione IRCCS Policlinico San Matteo, Pavia, Italy
Paola	Zelini	Fondazione IRCCS Policlinico San Matteo, Pavia, Italy
Piera	d’Angelo	Fondazione IRCCS Policlinico San Matteo, Pavia, Italy
Marica	De Cicco	Fondazione IRCCS Policlinico San Matteo, Pavia, Italy
Daniela	Cirasola	Fondazione IRCCS Policlinico San Matteo, Pavia, Italy
Federica	Zavaglio	Fondazione IRCCS Policlinico San Matteo, Pavia, Italy
Lea	Testa	Fondazione IRCCS Ca’ Granda Ospedale Maggiore Policlinico, Milano, Italy
Claudia	Ballerini	Fondazione IRCCS Ca’ Granda Ospedale Maggiore Policlinico, Milano, Italy
Rebecca	Stachetti	Fondazione IRCCS Ca’ Granda Ospedale Maggiore Policlinico, Milano, Italy
Marta	Ruggiero	Fondazione IRCCS Ca’ Granda Ospedale Maggiore Policlinico, Milano, Italy
Federica	De Liso	Fondazione IRCCS Ca’ Granda Ospedale Maggiore Policlinico, Milano, Italy
Annalisa	Cavallero	Università Milano-Bicocca Scuola di Medicina e Chirurgia, Fondazione Monza Brianza per il Bambino e la sua Mamma Onlus c/o Ospedale San Gerardo, Monza, Italy
Isadora	Vaglio Tessitore	Università Milano-Bicocca Scuola di Medicina e Chirurgia, Fondazione Monza Brianza per il Bambino e la sua Mamma Onlus c/o Ospedale San Gerardo, Monza, Italy
Maria Luisa	Ventura	Università Milano-Bicocca Scuola di Medicina e Chirurgia, Fondazione Monza Brianza per il Bambino e la sua Mamma Onlus c/o Ospedale San Gerardo, Monza, Italy
Mirko	Pozzoni	Gynecology and Obstetrics Department, IRCCS San Raffaele Hospital and University, Milan, Italy
Camilla	Merlo	ASST Spedali Civili di Brescia and University of Brescia, Italy
Giulia	Rivetti	ASST Spedali Civili di Brescia and University of Brescia, Italy
Vania	Spinoni	ASST Spedali Civili di Brescia and University of Brescia, Italy
Gaia	Belloni	ASST Fatebenefratelli-Sacco, Ospedale Macedonio Melloni, Milano, Italy
Camilla	Querzola	Ospedale dei Bambini Vittore Buzzi, Università di Milano, Milano, Italy

Marta	Pessina	Ospedale dei Bambini Vittore Buzzi, Università di Milano, Milano, Italy
Elisa	Ligato	Ospedale dei Bambini Vittore Buzzi, Università di Milano, Milano, Italy
Alice	Zavatta	Ospedale dei Bambini Vittore Buzzi, Università di Milano, Milano, Italy
Marta	Balconi	ASST Brianza (Ospedali di Carate e Vimercate), Italy
Serena	Mussi	ASST Brianza (Ospedali di Carate e Vimercate), Italy
Patrizia	Biraghi	ASST Brianza (Ospedali di Carate e Vimercate), Italy
Selene	Cammarata	Unit of Obstetrics and Gynecology, ASST Fatebenefratelli-Sacco, and Department of Biological and Clinical Sciences, University of Milan, Milan, Italy.
Sonia	Paolucci	Università dell'Insubria, Ospedale Del Ponte, Varese, Italy
Fabio	Ghezzi	Università dell'Insubria, Ospedale Del Ponte, Varese, Italy
Massimo	Agosti	Università dell'Insubria, Ospedale Del Ponte, Varese, Italy
Laura	Pellegrinelli	Dipartimento di Scienze Biomediche per la Salute, Università degli Studi di Milano, Italy
Cristina	Galli	Dipartimento di Scienze Biomediche per la Salute, Università degli Studi di Milano, Italy
Valeria	Primache	Dipartimento di Scienze Biomediche per la Salute, Università degli Studi di Milano, Italy

Study population, sample size and interim analysis

The study population was identified among immune pregnant women delivering in participating centers or being followed by outpatient clinics of participating centers in the first trimester of gestation. The expected rate of cCMV in immune mothers was conservatively estimated to be 0.4% on the basis of two previous studies conducted locally on newborns, without distinguishing between primary or non-primary maternal infection [1,2]. For Part 1 (epidemiology study) we planned to include in the final analysis 10,000 women with HCMV serology compatible with preconception immunity and with newborns examined for cCMV. With the planned sample size of 10,000 subjects, the 95% confidence interval (CI) of the expected incidence of congenital infection (0.4%) is 0.29-0.54%, corresponding to 29-54 newborns with documented cCMV.

As for Part 2, to demonstrate a 50% reduction of cCMV, i.e. from the expected 0.4% in the epidemiology study to 0.2% following information (80% power, alpha 5%), 13,523 pregnant women were needed. Sample size would have been either confirmed or recalculated based on results of an interim analysis planned after the examination of 5,000 newborns. In the event that ≤12 cases of cCMV were detected at the interim analysis (0.24%, 95% CI: 0.1-0.4%), Part 2 would have been cancelled: in such case, the expected 0.4% rate of cCMV in the uninformed population would fall outside of the 95% CI interval. Thus, a sample size larger than that planned would have been required to evaluate the effect of the behavioral intervention. However, had particular characteristics of transmitting women been identified in Part 1, sampling an enriched population would have been considered for Part 2 and the sample size recalculated accordingly.

Inclusion criteria

<u>Epidemiology study.</u>

- Adult (≥ 18 years old) pregnant women
- For women enrolled at ≤13 weeks gestation: presence of HCMV IgG and absence of IgM or presence of high avidity IgG with or without IgM
- For women enrolled at delivery only: presence of HCMV-specific IgG and absence of IgM or presence of high avidity IgG in case of positive IgM at ≤13 weeks gestation documented by medical report or by retrospective antibody determination on samples stored at ≤13 weeks
- Willingness to participate in the study
- Ability to understand information material
- Written informed consent

<u>Prevention study</u>

Same as for Epidemiology study excluding the option of enrollment at delivery.

Exclusion criteria

- Unreliable women as judged by the investigator
- Women not willing to give written consent

Laboratory testing

Dry newborn saliva swabs (FLOQSwabs, Copan, Brescia, Italy) were resuspended in 1ml PBS. After heating for 5' at 95°C, 5µl of the PBS resuspension were tested by real-time PCR (Artus CMV RG PCR kit; Qiagen). Preliminary experiments showed that the HCMV DNA load did not change significantly in dry saliva swabs maintained at room temperature for 7 days (Supplementary Figure 1). Previous studies reported similar results, showing that storage duration does not impact recovery efficiency [3]. Maternal dry saliva and vaginal swabs collected after delivery were also resuspended in PBS. The DNA was isolated with EZ1 DSP Virus Kit (Qiagen) from the maternal swab resuspension medium and maternal and newborn urine using the EZ1 Advanced XL instrument (Qiagen). The DNA was isolated from whole blood using QIAamp DNA Mini kit. HCMV-DNA was quantified by real-time PCR (Qiagen) using QiAgility and Rotor-Gene Q instruments (Qiagen).

Women with unknown serology prior to enrollment were tested for HCMV-specific IgG and IgM at each participating center. HCMV IgG avidity was tested in the central laboratory with LIAISON CMV IgG Avidity II (Diasorin, Saluggia, Italy).

Genotype-specific IgG response to gB and gH was performed as previously described [4]. An ELISA was used to detect peptide-specific IgG antibodies capable of recognizing linear peptides near the N-termini of the proteins encoded by the gB1, gB2/3, gB4, and gH1, gH2 genotypes. For gB, genotypes 2 and 3 cannot be distinguished in the N-terminus region and were considered collectively as gB2/3. Three peptides of 20 aa (overlapping by 10-13 aa) were used for each gB genotype and one peptide of 20 aa was used for gH genotype. Half-area 96-well microplates were coated with streptavidin (0.1 M) diluted in bicarbonate buffer. After a block with 5% (wt/vol) skimmed milk, biotinylated peptides (170 nM) were immobilized in the wells. After washing, the plates were incubated with human serum (diluted 1:50), then with horseradish peroxidase-labeled goat IgG to human IgG and, finally, with 30mg/ml orthophenylendiamine before the addition of 4 N sulphuric acid. The optical density (OD) value of the serum incubated without peptide was subtracted from the OD value of the serum incubated with peptide. Mean OD values were calculated from triplicate assays. Results above 0.3 OD in at least one of the peptides tested were considered positive.

1. Giaquinto C, Falconi P, Bellattato M et al. L'infezione congenita da CMV: studio prospettico. Rivista di Infettivologia Pediatrica, 1988;4:219-223.
2. Barbi M, Binda S, Caroppo S, et al. Multicity Italian study of congenital cytomegalovirus infection. Ped Infect Dis J, 2006;25:156-159.
3. Kohmer N, Nagel A, Berger A, et al. Laboratory diagnosis of congenital CMV infection in newborns: Impact of pre-analytic factors. J Clin Virol. 2019;115:32-36.
4. Zavaglio F, Fiorina L, Suárez NM, et al. Detection of Genotype-Specific Antibody Responses to Glycoproteins B and H in Primary and Non-Primary Human Cytomegalovirus Infections by Peptide-Based ELISA. Viruses, 2021;13:399.

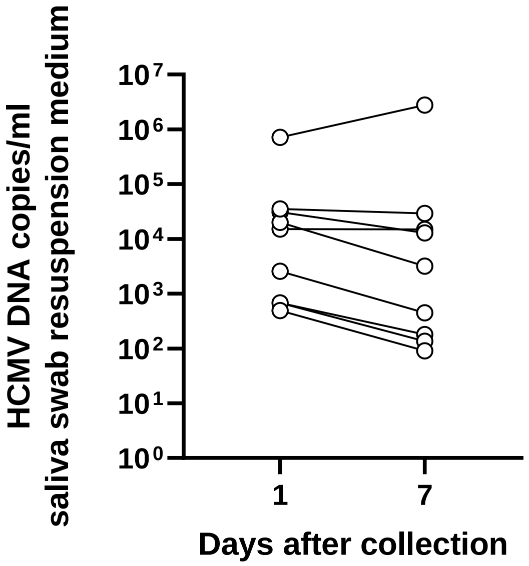

Supplementary Figure 1. HCMV DNA level in saliva swabs maintained at room temperature and tested at day 1 and day 7 after collection.

Supplementary Table 1. Results of HCMV DNA screening and confirmatory tests in the 45 newborns with positive saliva screening.

Subject	Real-time PCR screening on saliva		Confirmation in second real-time PCR on saliva	Real-time PCR on urine		Real-time PCR on dry blood spot	Newborn's diagnosis
	Result	Copies/ml		Result	Copies/ml		
363-921	positive	149,950	yes	positive	1,496,529	ND	infected
363-999	positive	169,333	yes	positive	7,346,775	ND	infected
364-284	positive	48,941	yes	positive	32,802,624	ND	infected
365-63	positive	2,147,137	yes	ND	ND	positive	infected
365-81	positive	1,359	yes	positive	3,324,726	positive	infected
367-145 Twin 1	positive	1,491	yes	positive	97,562	ND	infected
367-145 Twin 2	positive	512	yes	positive	55,690	ND	infected
367-227	positive	1,324	yes	positive	107,738	ND	infected
368-1103	positive	121,212	yes	positive	3,827,428	ND	infected
369-143	positive	5,215	yes	positive	111,285,558	ND	infected
369-156	positive	4,749	yes	positive	4,161	ND	infected
369-79	positive	406,408	yes	positive	484,721	ND	infected
371-1965	positive	3,241,918	yes	positive	60,199,569	ND	infected
371-403 Twin 2	positive	72,425	yes	positive	36,460	ND	infected
371-426	positive	86,571	yes	positive	50,351	ND	infected
372-470	positive	35,566	yes	positive	1,423	ND	infected
373-516	positive	55,634	yes	positive	41,026,281	ND	infected
363-669	positive	248	yes	negative	0	ND	non-infected
369-103	positive	106	yes	negative	0	ND	non-infected
371-1194	positive	92	yes	negative	0	ND	non-infected
371-152	positive	19	yes	negative	0	ND	non-infected
371-1787	positive	35	yes	negative	0	ND	non-infected
371-1788	positive	39	yes	negative	0	ND	non-infected
371-2919	positive	51	yes	negative	0	ND	non-infected
371-323	positive	7	yes	negative	0	ND	non-infected
371-403 Twin 1	positive	8	yes	negative	0	ND	non-infected
371-860	positive	15	yes	negative	0	ND	non-infected
373-23	positive	25	yes	negative	0	ND	non-infected
363-327	positive	28	No	ND	ND	ND	non-infected
363-879	positive	14	No	ND	ND	ND	non-infected
371-117	positive	5	No	ND	ND	ND	non-infected
371-135	positive	5	No	ND	ND	ND	non-infected
371-175	positive	9	No	ND	ND	ND	non-infected
371-1875	positive	8	No	ND	ND	ND	non-infected
371-2461	positive	18	No	ND	ND	ND	non-infected
371-2580	positive	10	No	ND	ND	ND	non-infected
371-276	positive	18	No	ND	ND	ND	non-infected
371-2873	positive	18	No	ND	ND	ND	non-infected
371-2935	positive	10	No	ND	ND	ND	non-infected
371-349	positive	8	No	ND	ND	ND	non-infected
371-374	positive	7	No	ND	ND	ND	non-infected
371-548	positive	19	No	ND	ND	ND	non-infected
371-586	positive	16	No	ND	ND	ND	non-infected
372-78	positive	24	No	ND	ND	ND	non-infected
373-936	positive	8	No	ND	ND	ND	non-infected

ND, not done

Supplementary Table 2. Genotype-specific anti-gB and anti-gH IgG antibody response

Subject	gB-type		gH-type	
	<13 weeks	Delivery	<13 weeks	Delivery
SR 373-516	undetectable	undetectable	gH2	gH2
PV 363-921	gB2/3	gB2/3	gH1	gH1
BU 364-284	gB2/3	gB2/3	gH2	gH2
VI 369-143	undetectable	**gB2/3**	gH1, gH2	gH1, gH2
PV 363-999	gB2/3	gB2/3	gH1	gH1
PV 363-1104	undetectable	undetectable	undetectable	undetectable
SC 372-1965	undetectable	undetectable	gH2	gH2

Serum samples were tested for the presence of IgG antibodies against the following gB and gH genotypes: gB1, gB2/3, gB4, gH1, gH2. The genotype-specific antibody responses detected are shown. The appearance of a novel gB genotype-specific IgG antibody is shown in bold character.

Supplementary Table 3. Rate of congenital cytomegalovirus infection in immune mothers from different countries.

Country (seroprevalence)	No. newborns screened	No. [% (95% CI)] of infected newborns	No. (%) of symptomatic newborns	Study*
Brazil (98%)	1,685	8 [0.47 (0.21-0.93)]	0/8 (0)	Mussi-Pinhata et al., 2018 [16]
France (61%)	1,454	3 [0.21 (0.04-0.60)]	Not reported	Leruez-Ville et al., 2017 [20]
Italy (68%)	9,662	18 [0.19 (0.11-0.29)]	3/18 (16.7)	CHILd

*Numbers in brackets indicate the reference number in the “References” section of the main text
